# Inhibition of Hepatitis E Virus Spread by the Natural Compound Silvestrol

**DOI:** 10.3390/v10060301

**Published:** 2018-06-02

**Authors:** Mirco Glitscher, Kiyoshi Himmelsbach, Kathrin Woytinek, Reimar Johne, Andreas Reuter, Jelena Spiric, Luisa Schwaben, Arnold Grünweller, Eberhard Hildt

**Affiliations:** 1 Department of Virology, Paul-Ehrlich-Institut, 63225 Langen, Germany; mirco.glitscher@pei.de (M.G.); kiyoshi.himmelsbach@pei.de (K.H.); kathrin.woytinek@pei.de (K.W.); 2 Federal Institute for Risk Assessment, 10589 Berlin, Germany; Reimar.Johne@bfr.bund.de; 3 Department of Allergology, Paul-Ehrlich-Institut, 63225 Langen, Germany; Andreas.Reuter@pei.de (A.R.); Jelena.Spiric@pei.de (J.S.); Luisa.schwaben@pei.de (L.S.); 4 Institute of Pharmaceutical Chemistry, Philipps-Universität Marburg, 35037 Marburg, Germany; arnold.gruenweller@staff.uni-marburg.de; 5 German Center for Infection Research (DZIF), 38124 Braunschweig, Germany

**Keywords:** HEV, silvestrol, antiviral, eIF4A, MVP

## Abstract

Every year, there are about 20 Mio hepatitis E virus (HEV) infections and 60,000 deaths that are associated with HEV worldwide. At the present, there exists no specific therapy for HEV. The natural compound silvestrol has a potent antiviral effect against the (−)-strand RNA-virus Ebola virus, and also against the (+)-strand RNA viruses Corona-, Picorna-, and Zika virus. The inhibitory effect on virus spread is due to an inhibition of the DEAD-box RNA helicase eIF4A, which is required to unwind structured 5′-untranslated regions (UTRs). This leads to an impaired translation of viral RNA. The HEV (+)-strand RNA genome contains a 5′-capped, short 5′-UTR. This study aims to analyze the impact of silvestrol on the HEV life cycle. Persistently infected A549 cells were instrumental. This study identifies silvestrol as a potent inhibitor of the release of HEV infectious viral particles. This goes along with a strongly reduced HEV capsid protein translation, retention of viral RNA inside the cytoplasm, and without major cytotoxic effects. Interestingly, in parallel silvestrol affects the activity of the antiviral major vault protein (MVP) by translocation from the cytoplasm to the perinuclear membrane. These data further characterize the complex antiviral activity of silvestrol and show silvestrol’s broad spectrum of function, since HEV is a virus without complex secondary structures in its genome, but it is still affected.

## 1. Introduction

The Hepatitis E Virus, which was first discovered in 1980 [1], is one of the major causes of an acute hepatitis worldwide, most often spreading in developing countries with poor hygiene standards, although hepatitis E virus (HEV) immune responses can be observed all over the world [2]. Especially in recent years, since more case studies and controls have been conducted, it has been observed that HEV is also widespread in European countries [3] and in the Americas [4,5]. Since this topic has become more aware, it became obvious that HEV is not only a virus that is considered to be water-borne, but also food-borne [6]. Livestock, for example, domestic pigs, can be infected with zoonotic genotypes, making them possible conductors and transmitters for HEV infections. In the past decade, over 21,000 new infections were registered in Europe, showing a 10-fold increase in the number of cases. Not only meat-consuming customers in Western countries are at risk of HEV infections, but also patients in need of blood-transfusion [7]. The clinical course of hepatitis developed upon HEV infection bears a mortality rate of 1% in healthy adults, whereas fatality increases to 20% in pregnant women that were infected with genotype 1 [8]. Chronic HEV infections, which may develop to liver cirrhosis, are increasingly recognized in immunosuppressed transplant patients [9,10].

HEV is classified as a (+)-strand ssRNA virus and a member of the *Hepeviridae* family. Four major human-pathogenic genotypes have been identified, with genotype 1 and 2 being restricted to humans, whereas genotype 3 and 4 are able to infect both human and swine. All genotypes contain a ~7.4 kb genome [11], which is composed of three open reading frames (ORF): *ORF1* encodes a non-structural polyprotein (pORF1), which is mainly responsible for efficient virus replication, *ORF2* encodes the capsid-forming core protein (pORF2), and *ORF3* is the coding region for a protein of unknown function (pORF3) [12]. HEV pORF1 is the only polyprotein that is found in the virus’ proteome, which is composed of four different subdomains: a methyltransferase domain, a papain-like cysteine protease domain, an RNA helicase domain, and an RNA dependent RNA polymerase domain, as indicated by homology analyses [13]. There is evidence that this polyprotein is further processed and cleaved into smaller proteins by a cysteine protease, with each fragment showing the proposed catalytic activities [14]. The viral RNA genome further contains a 5′-capped, short 5′-UTR (~26 base pairs) [15]. This indicates a dependency on cap-recognizing proteins and a possible regulation that is based on the untranslated region, since the genome itself serves as a template for protein biosynthesis. Moreover, 3′-poly adenylation is found in the virus’ genome. Additional studies on the HEV genome showed that not only the full genome itself serves as the sole template for viral protein synthesis, but also a bicistronic, subgenomic RNA coding for pORF2, and pORF3 [16,17]. HEV egress, after capsid assembly, is then managed via the exosomal pathway [18], with pORF3 being an important interaction partner of tumor susceptibility gene 101 (TSG101) [19], possibly tethering the capsid to the endosomal sorting complexes that are required for transport (ESCRT) that mediate the entry into the multivesicular bodies (MVBs). Therefore, viral particles are found as quasi-enveloped particles (surrounded by an exosomal membrane) in both cell culture supernatant and patient serum, while being excreted (via feces) as naked capsid viral particles [18,20,21]. Just recently, a form of pORF2 has been described to be secreted as homodimers, in addition to the population found in assembled capsids [22].

Silvestrol, which is a cyclopenta[b]benzofuran, is a natural compound that is extracted from the plant species *Aglaia foveolata* [23]. This compound is a potent and selective inhibitor of the eukaryotic initiation factor 4A (eIF4A), an RNA helicase that is required to unwind RNA secondary structures in the 5′-UTRs of mRNAs, thus creating a binding platform for the 43S preinitiation complex. As such, it was first described as a growth-inhibiting agent in human breast and prostate xenograft models by inhibiting translation initiation from 5′-m7GTP capped mRNAs with extended and structured 5′-UTRs, as often found in proto-oncogenes, while being well tolerated by the mice used in the experiments [24]. Furthermore, silvestrol prolongs the survival rate of mice with hepatocellular cancer, and therefore is discussed as a potential, novel anticancer drug [25], although it has not been used in human clinical trials so far. Just recently, a study has been published showing a potent antiviral effect of silvestrol in cells that are infected with the Ebola virus [26], a (−)-strand ssRNA virus that transcribes from its genomic RNA 5′capped mRNAs with relatively long and structured 5′-UTRs, which seemed to be causative for the compound’s effect. Other examples for RNA viruses being affected by silvestrol were identified as Coronavirus (CoV), human rhinovirus (HRV) A1, Zika virus (ZIKV), and poliovirus type 1 (PV) [27,28]. Therefore, silvestrol appeared as an interesting agent to be tested on a (+)-strand ssRNA virus containing only a short 5′-UTR, such as HEV.

In the course of the study, the Major Vault Protein (MVP) has been discovered to be of a certain importance in the context of HEV and silvestrol. MVP is a 100 kDa cytosolic protein and has been described to build up the main part of ribonucleoprotein particles, called vaults [29]. It has been linked to the drug-resistance of several types of non-viral diseases [30,31,32], as well as to virus-mediated pathogenesis [33] modulating innate antiviral immune-responses [34,35].

To gain insights into the effects of silvestrol on the HEV life cycle, a persistently HEV-infected A549 cell line was used. This specific cell line is infected with an HEV isolate from a chronically infected patient, the HEV genotype 3 strain 47832c, and shows a long-lasting HEV gene-expression and viral particle production, which is optimal for experimental usage [36].

The aim of this study is to evaluate the possible antiviral effects of silvestrol on HEV, thereby getting deeper insights in the virus’ life cycle and to further characterize silvestrol with respect to its antiviral activity, possibly identifying novel host-factors being important in the context of HEV-infection besides eIF4A.

## 2. Materials and Methods

### 2.1. Cell Culture

A549 cells (German Collection of Microorganisms and Cell Cutures, DSMZ no.:ACC 107) originate from a human lung carcinoma. A549/D3 cells represent a subclone of A549 cells (ATCC CCL-185) that showed increased susceptibility to infection with HEV strain 47832c (genotype 3c) [37]. This HEV genotype 3 strain has been originally isolated from a chronically infected patient [36]. A cell line that was persistently infected with strain 47832c was generated by the inoculation of A549 cells with the virus and subsequent cell passaging [36]. This cell line shows long-term expression of HEV proteins and the release of infectious viral particles [36]. A549/D3 cells, as well as persistently HEV-infected A549 cells, were cultured in High-Glucose (4.5 g/L) Dulbecco’s Modified Eagle’s Medium (DMEM, BioWest, Nuaillé, France), supplemented with 2 mM l-glutamine, 100 µg/mL streptomycin and 100 U/mL penicillin (PAA, Linz, Austria), and 10% (*v*/*v*) fetal bovine serum (FBS superior; Sigma-Aldrich, Hamburg, Germany). Cells were cultured at 37 °C with a humidified atmosphere containing 5% CO_2_.

### 2.2. Treatment with Silvestrol

Cells were seeded in polystyrene 6-well plates or 12-well plates (Greiner, Frickenhausen, Germany) containing 3.5 × 10^5^ cells/well or 5 × 10^4^ cells/well, respectively.

After 24 h, the culture medium was exchanged against medium containing 2 nM,10 nM or 50 nM silvestrol (originating from Medchemexpress LLc, Princeton, NJ, USA; purity >98%) or 0.1% (*v*/*v*) DMSO (Genaxxon, Biberach, Germany). Each 24 h, the medium was either exchanged against fresh treatment medium or harvested for the respective time points.

### 2.3. SDS-PAGE and Western Blot

Cells were lyzed with RIPA buffer (50 mM Tris, 150 mM NaCl, 0.1% (*w*/*v*) SDS, 0.5% (*w*/*v*) sodium deoxycholate, 1% (*w*/*v*) Triton X-100, pH 7.2), sonicated, and boiled in gel loading buffer (4% (*w*/*v*) SDS, 125 mM Tris, 10% (*v*/*v*) glycerol, 10% (*v*/*v*) 2-mercaptoethanol, 0.02%, pH 6.8; 4× buffer). The SDS-PAGE was performed, as described before [38]. Proteins that were separated by SDS-PAGE were blotted on a PVDF-membrane by applying a current of 1.5 mA/cm^2^ gel using a semi-dry blotting approach, as described [39].

Proteins of interest were detected with specific primary antibodies after blotting (rabbit-anti-HEV capsid protein, antiserum 8282 kindly provided by Dr. Rainer Ulrich, Friedrich-Loeffler-Institute, Federal Research Institute for Animal Health, Grifswald, Germany). Beta-actin was detected using a commercially available monoclonal mouse antibody (mouse anti beta-actin, 2 mL, Sigma-Aldrich, Hamburg, Germany). MVP was detected using a commercially available monoclonal mouse antibody (mouse anti LRP, 1032, SantaCruz Biotechnology, Heidelberg, Germany), GAPDH has been detected with a polyclonal antiserum (rabbit-anti GAPDH (FL335), Santa Cruz Biotechnology, Heidelberg, Germany), and Histone H2B has been detected with a polyclonal rabbit antiserum (rabbit-anti-Histone H2B, FL126, Santa Cruz Biotechnology, Heidelberg, Germany). Secondary antibody detection was performed using the LI-COR Odyssey Infrared Imager (Biosciences, Lincoln, NE, USA), according to the manufacturer’s protocol. Secondary antibodies were obtained from LI-COR. Total protein stain was performed using Ponceau S (Carl Roth, Karlsruhe, Germany).

### 2.4. RNA Extraction and cDNA-Synthesis

Cells were lyzed and RNA was extracted using PeqGOLD TriFast (PeqLab, Erlangen, Germany). Reverse transcription from extracted RNA was performed using RevertAid H Minus RT (Thermo-Scientific, Braunschweig, Germany). RNA from cell culture supernatants was purified using the QIAmp Viral RNA Kit (QIAGEN, Hilden, Germany). All of the procedures were performed, according to the manufacturer’s protocols.

### 2.5. qPCR and RT-qPCR

Quantification of intracellular viral RNA was performed based on a SYBR-Green system using the Maxima SYBR-Green qPCR Kit (Thermo-Scientific, Braunschweig, Germany) with primers flanking HEV ORF2 (FWD: 5′-GGT GGT TTC TGG GGT GAC-3′; REV: 5′-AGG GGT TGG TTG GAT GA-3′) and RPL27 as a housekeeping gene (FWD: 5′-AAA GCT GTC ATC GTG AAG AAC -3′; REV: 5′-GCT GCT ACT TTG CGG GGG TAG-3′). In order to be able to normalize intracellular measurements, SYBR-Green based assays were used for this experimental setup. Quantification of extracellular viral RNA was performed based on a hydrolysis-probe system using the LightCycler Multiplex RNA Master Mix (Roche Diagnostics, Mannheim, Germany) and LightMix Modular Hepatitis E Virus Kit (quantitative kit, TIB Molbio, Berlin, Germany). Light mix modular HEV kit is a CE marked based real time PCR kit with a detection limit of 200 IU/mL for HEV genotypes 1–4 (containing HEV specific primers and a respective hydrolysis probe). All of the procedures were performed according to the manufacturer’s protocols and were analyzed with either LightCycler 2.0 or LightCycler 480 Instrument II (Roche, Mannheim, Germany).

### 2.6. Tissue Culture Infective Dose (TCID_50_)

A549/D3 cells were seeded in flat-bottom polystyrene 96-well plates (Greiner, Frickenhausen, Germany) with a density of 1 × 10^4^ cells/well. Five hours post seeding, cells were infected with supernatants harvested 24 h, 48 h, and 72 h after the beginning of the silvestrol treatment. Dilution-series were used ranging from undiluted to 1:100,000, with a dilution factor of 10 for each step, to fit the aims of an End Point Dilution Assay (EPDA). Infection lasted for 16 h and was stopped by a medium exchange. Cells were cultured for 48 h post infection and infection was evaluated via immunofluorescence. TCID_50_ values were calculated as described before [40].

### 2.7. Luciferase Assay

A549 cells were transfected (electroporation) with an in vitro transcribed HEV reporter construct, bearing the coding sequence for the secreted *Gaussia* luciferase inserted into HEV ORF2 of HEV genotype 3 strain p6Kernow, which was kindly provided by Prof. Suzanne Emmerson, as described previously [41]. Lysates were harvested 24 h, 48 h, and 72 h after the beginning of the silvestrol treatment and were monitored for their luciferase activity using the Gaussia-Juice Kit (pjk, Kleinbittersdorf, Germany), according to the manufacturer’s protocol. Chemiluminescence was measured with an Orion II Microplate Luminometer (Titertek, Pforzheim, DE, Germany). Values were normalized to total protein amounts of respective cells. Protein amounts were measured using a Bradford Assay, according to the manufacturer’s protocol (Bradford Reagent, Thermo-Scientific, Braunschweig, Germany).

### 2.8. Viability Assay

Cells were seeded in flat-bottom polystyrene 96-well plates (Greiner, Frickenhausen, Germany) with a density of 1 × 10^4^ cells/well. Viability was monitored 24 h, 48 h, and 72 h after the beginning of the silvestrol treatment using the lactate dehydrogenase (LDH) Cytotoxicity Detection Kit (Clontech, Mountain View, CA, USA) and the PrestoBlue^®^ Cell Viability Reagent (Life Technologies, Carlsbad, CA, USA), according to the manufacturer’s protocol. Absorbance and fluorescence were measured with a Tecan Microplate Reader (Tecan Group, Männedorf, Switzerland).

### 2.9. Immunofluorescence Microscopy

Cells were fixed with 100% ethanol, blocked with 10% (*w*/*v*) bovine serum albumin in TBS-T (Tris buffered saline (20 mM Tris, 150 mM sodium chloride, pH 8.8), supplemented with 0.05% (*v*/*v*) Tween 20) and incubated with a respective primary antibody (monoclonal mouse-anti-HEV capsid protein, 5G5, kindly provided by Prof. Jihong Meng or monoclonal mouse-anti-LRP(MVP), 1014, SantaCruz Biotechnology, Heidelberg, Germany). Primary antibody epitopes were detected with AlexaFluor488 or AlexaFluor546 coupled donkey-anti-mouse or donkey-anti-rabbit antibodies (Invitrogen, Carlsbad, CA, USA). Nuclei were stained with 250 ng/mL 4′,6-Diamidin-2-phenylindole (DAPI) in secondary antibody solution. Microscopy was performed using a Nikon Ti-U E20L80 microscope (Nikon Metrology GmbH, Alzenau, Germany) and imaging was performed using a Mono DS-Qi2 camera (Nikon Metrology GmbH, Alzenau, Germany). For confocal microscopy, a Confocal Laser Scanning Microscope 510 meta (Carl Zeiss AG, Jena, Germany) has been used.

### 2.10. In-Solution Protein Digestion and LC/MS Analysis

Persistently HEV-infected A549 cells and A549/D3 cells were cultured for 24 h in the presence or absence of 10 nM silvestrol. Cells were harvested, homogenized (in PBS), and cytosolic proteins were extracted with sequential centrifugation in a Beckmann Optima XPN-80 ultracentrifuge (Beckmann Coulter, Krefeld, Germany), as described previously. Absence of nuclear proteins in the cytosolic fraction was tested by western blot analysis using Histone [42].

The study design included three treatment replicates for each cell type, and every replicate was subjected to LC/MS analysis, recording 12 data sets in total. Prior to in-solution digestion assay, the protein concentration of each sample was determined by the Bradford Assay. Ten micrograms of the total soluble protein were adjusted to a total volume of 30 μL in 50 mM NH_4_HCO_3_. The samples were reduced with 12.5 μL of 40 mM DTT in 50 mM NH_4_HCO_3_ for 15 min at 60 °C in a thermal cycler (Bibby Scientific, Staffordshire, UK) and alkylated with 12.5 μL of 218 mM iodoacetamide in 50 mM NH_4_HCO_3_ for 15 min at ambient temperature in the dark. The samples were digested with trypsin at 1:8 protease to protein ratio (5 μL of 0.250 μg/μL in 25 mM NH_4_HCO_3_) and were incubated for 16 h at 37 °C in a thermal cycler. The reaction was stopped by the addition of 5% formic acid (6.5 μL). The resulting tryptic digests were stored at −80 °C until further MS analyses.

Each digested sample was spiked with MassPREP ADH digestion standard (Waters, Manchester, UK) that was used as an internal standard at a level of 20 fmol per μL. For a sample analysis, 670 ng of digested protein aliquots were analyzed using a nano-UPLC ESI Q-TOF-MS (nanoACQUITY UPLC M-Class System and Synapt G2 MS; Waters, Milford, CT, USA) equipped with a C18 trap column (ACQUITY UPLC M-Class Symmetry C18 Trap Column, 100 Å, 5 µm, 180 µm × 20 mm; Waters, Milford, CT, USA) and a HSS T3 analytical column (nanoACQUITY UPLC HSS T3 Column, 1.8 µm, 75 µm × 150 mm Waters, Milford, CT, USA). The MS was operated in positive V-mode using standard parameters and lockmass calibrated (Glu-1-Fibrinopeptide 0.1 pmol/μL, at 0.5 μL/min, 1 scan every 20 s). Data were acquired using data-independent acquisition (UDMS^E^) mode [43], altering between low (0 V) and high (ramped from 17–45 V and 45–60 V) collision energies, with a scan time of 0.5 s. The data were acquired in m/z range from 100 to 2000. The raw data processing, database search (database consisting of all unreviewed UniProt entries for viruses (taxon identifier 10,239), Homo sapiens (9606) and bos (9903) available as of August 2017), protein quantification, and statistical evaluation of the semi-quantitative protein expression profiles were performed with Progenesis QI for Proteomics (Waters, Manchaster, UK).

### 2.11. Statistical Analysis

All of the statistical analyses, but the ANOVA test of the MS data, were performed with Prism GraphPad 7.0, using multiple t-tests for determination of *p*-values. Error bars are displayed as value ± SEM. Single datasets were always referred to their DMSO control, setting the control to 1.0, respectively, and subsequently pooled to yield a mean value over all experiments of one kind. As the control groups (DMSO) were arbitrarily set as 1, a standard deviation for the control groups cannot be reported, as the standardization of the measured values (relative to the control group) was performed for each of the independent assays. Therefore, measurements for the treatment groups in each assay were dependent (matched). N = x always displays the number of separately performed, independent experiments.

## 3. Results

### 3.1. Silvestrol Causes a Robust Reduction in the Number of Released HEV Particles

**Figure 1:**
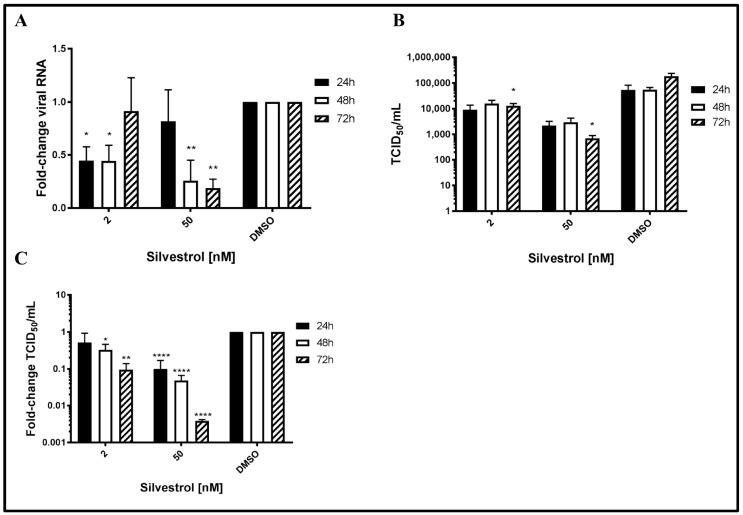
Repressive effect of silvestrol on release of infectious viral particles from A549 cells persistently infected with hepatitis E virus (HEV) strain 47842c. (**A**) qPCR quantification of extracellular viral RNA in the supernatant derived from silvestrol treated cells (2 nM and 50 nM). The values were referred to the DMSO control; (**B**) Absolute tissue culture infective dose (TCID_50_) values determined after A549/D3 infection (logarithmic scale); (**C**) Relative TCID_50_ values compared to DMSO on the basis of (**B**). *n* = 3 for all experiments; * *p* < 0.05, ** *p* < 0.01, **** *p* < 0.0001.

To study the potential antiviral effect of silvestrol on HEV, persistently HEV-infected A549 cells were treated with silvestrol and the number of released HEV genomes was analyzed by RT-qPCR. For silvestrol concentrations of 2 nM, extracellular RNA levels significantly decreased after 24 h and 48 h of treatment as compared to DMSO (more than 2-fold). However after 72 h, no significant reduction of viral RNA in the cell culture supernatant was observed. In contrast to this, for cells treated with 50 nM silvestrol, no significant change in the number of HEV-specific genomes was observed after 24 h (Figure 1A). Nonetheless, after 48 h and 72 h, a robust reduction of extracellular HEV RNA (up to five-fold as compared to DMSO) was found.

As quantification of viral genomes does not directly reflect the number of infectious viral particles [44,45], determination of TCID_50_ was performed to directly quantify the infectious viral particles in the cell culture supernatant. Cell culture supernatants of DMSO treated cells served as a control. Viral titers in cell culture supernatants were analyzed via EPDA and subsequent TCID_50_ determination (with Figure 1B showing absolute values and Figure 1C showing relative quantifications of 1B).

In contrast to the effect on the amount of viral RNA, a significant reduction in the release of infectious viral particles for the treatment with both 2 nM and 50 nM silvestrol was determined for all time points. The decrease ranges from a 2- to 10-fold reduction for 2 nM and from a 3- to 250-fold reduction for 50 nM silvestrol (Figure 1B,C). In both cases, the decrease becomes more pronounced with increasing treatment times.

Taken together, these data indicate that silvestrol causes a significant decrease in the number of released infectious viral particles.

### 3.2. Decreased Number of Released Viral Particles Is Not Caused by a General Cytotoxic or Cytostatic Effect of Silvestrol

**Figure 2:**
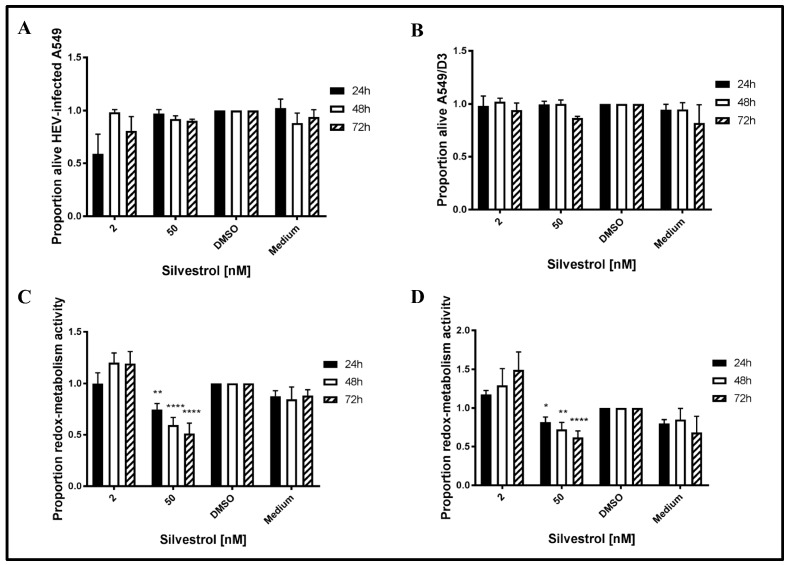
Lactate dehydrogenase (LDH) and PrestoBlue assays to determine silvestrol-mediated cytotoxicity and cytostaticity. (**A**,**B**) Processed data for LDH-activity in cell-culture supernatants derived from cells treated with 2 or 50 nM silvestrol as compared to respective DMSO controls in (**A**) A549 cells persistently infected with HEV strain 48932c or (**B**) in non-infected A549/D3. (**C**,**D**) Processed data for redox-metabolic activity in cells treated with 2 or 50 nM silvestrol when compared to respective DMSO controls in (**C**) A549 cells persistently infected with HEV strain 47832c or (**D**) in non-infected A549/D3 cells. * *p* < 0.05, ** *p* < 0.01, **** *p* < 0.0001.

To exclude that the observed effects of silvestrol on HEV release are due to a cytotoxic effect, the redox-metabolic activity of silvestrol-treated cells was analyzed by PrestoBlue assays and the cellular integrity was assessed by LDH assays. To analyze, whether HEV replication sensitizes cells for silvestrol-mediated toxic effects, non-infected A549/D3 cells were tested in addition (Figure 2).

The LDH assays for 2 nM silvestrol show that cellular integrity is stronger affected after 24 h of treatment, as compared to the incubation periods of 48 h and 72 h. Moreover, it has been found that a higher concentration (50 nM) does not lead to an increased cell death at the investigated time points (Figure 2A,B). Time points were chosen to be in concordance with the overall experimental setup.

As reflected by the PrestoBlue assay, which was used to evaluate the redox-metabolic activity (Figure 2C,D), silvestrol does not significantly affect the redox-metabolic activity of the persistently HEV-infected A549 cells in the lower concentration (2 nM). However, at 50 nM, a reduction is observed in both cell lines (Figure 2C,D). As described above, the reduced redox-metabolic activity is not associated with a significantly elevated cell death, as shown by the LDH assay (Figure 2A,B).

This shows that the antiviral effect of silvestrol at 50 nM (up to 250-fold) is not due to an effect on cellular integrity, as reflected by the results of the LDH assay. 

### 3.3. Elevated Levels of HEV-Specific RNA Are Found in Cells Treated with Lower Concentrations of Silvestrol

The next set of experiments was performed to monitor the effects of silvestrol on the level of intracellular HEV-specific RNA. Therefore, total RNA was extracted from persistently HEV-infected A549 cells at 24 h, 48 h, and 72 h after beginning of the silvestrol treatment and analyzed by qPCR. Primers used for this experiment are flanking the ORF2 region of the viral genome, thereby quantifying both genomic and subgenomic RNA.

**Figure 3:**
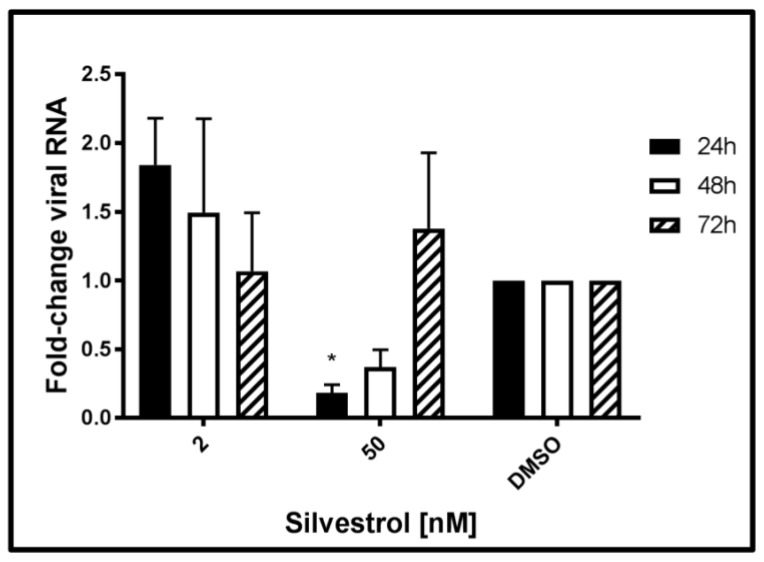
Effect of silvestrol on the intracellular amount of HEV-specific RNA in A549 cells persistently infected with HEV strain 47842c. qPCR analysis using ORF2-specific primers of total RNA isolated from A549 cells persistently infected with HEV; data were normalized to intracellular RPL27 RNA levels. Cells were treated with 2 nM or 50 nM silvestrol, DMSO treatment served as control. *n* = 3; * *p* < 0.05.

The qPCR (Figure 3) revealed that treatment with silvestrol at concentration of 2 nM led to an initial increase (24 h) of intracellular HEV-specific RNA as compared to the control, which was not significant. Treating cells with 50 nM silvestrol showed a significant decrease in the intracellular amount of viral RNA for 24 h and 48 h. However, after 72 h, the intracellular amount of HEV-specific RNA was almost unchanged as compared to the control.

Taken together, these data show that the effect of silvestrol on the intracellular amount of HEV-specific RNA depends on the applied silvestrol concentration and on the duration of the treatment. This might reflect the overlapping effects of silvestrol, at least on viral RNA stability, translation of viral RNA, viral replication, and viral release.

### 3.4. Silvestrol Reduces the Intracellular Amount of HEV Capsid Protein

The increase of intracellular HEV-RNA in silvestrol-treated, persistently HEV-infected cells might have several reasons. One hypothesis is that, due to impaired packaging of viral genomes into capsids, fewer particles can be formed and released, leading to an intracellular accumulation of the viral RNA.

**Figure 4:**
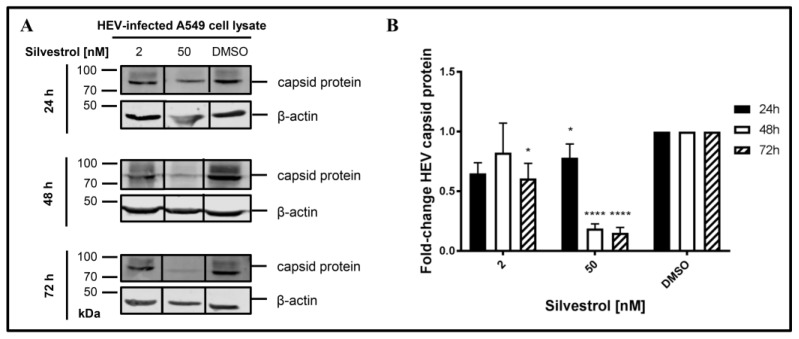
Effects of silvestrol on intracellular HEV capsid protein levels in A549 cells persistently infected with HEV strain 47832c. (**A**) Representative Western Blot of cellular lysates derived from cells treated with either 2 nM or 50 nM silvestrol. DMSO treatment served as control. For detection, a capsid protein specific antiserum and a beta-actin-specific antibody (loading control) were used; (**B**) Relative quantification of Western Blot band intensities normalized to intracellular beta-actin levels referred to the DMSO control. *n* = 3; * *p* < 0.05, **** *p* < 0.0001.

To experimentally control this hypothesis, the amount of intracellular capsid protein in persistently HEV-infected A549 cells that were treated with silvestrol for different time points was determined by Western Blot analyses (Figure 4).

Indeed, a decrease in the intracellular amount of HEV capsid protein levels was observed for 50 nM silvestrol after treatment for 24 h, 48 h, and 72 h and for 2 nM after 72 h of treatment (Figure 4B).

**Figure 5:**
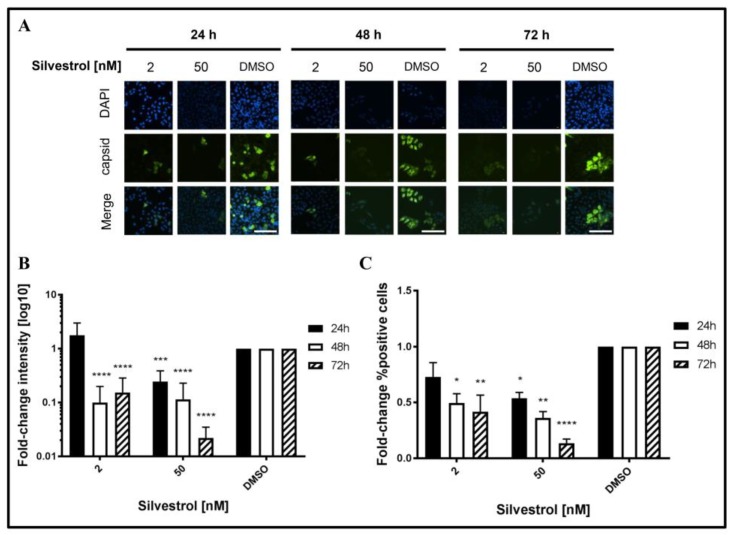
Immunofluorescence staining of silvestrol-treated A549 cells persistently infected with HEV strain 47832c. (**A**) Persistently HEV-infected A549 cells were seeded on cover-slips and treated with respective concentrations of silvestrol for the indicated time points. Nuclei were stained using 4′,6-Diamidin-2-phenylindole (DAPI) (blue) and an anti-HEV capsid protein (core) antiserum (green), scale bar =50 µm (**B**) Fluorescence intensity quantification of HEV capsid protein immunostaining and referred to the respective DMSO control to gain fold-change; and, (**C**) Comparison of the proportion of HEV capsid protein expressing cells depicted in Figure 5A referred to the respective DMSO control. *n* = 4 for all experiments; * *p* < 0.05, ** *p* < 0.01, *** *p* < 0.001, **** *p* < 0.0001.

This was confirmed by quantitative immunofluorescence microscopy of silvestrol-treated persistently HEV-infected A549D3 cells using a capsid protein-specific antiserum (Figure 5A) and comparing the respective quantifications to the DMSO control (Figure 5B,C). 

The fluorescence intensity of the HEV capsid protein expressing cells decreased over time for concentrations of 2 nM and 50 nM silvestrol, as compared to the control (Figure 5B). For the proportion of HEV-positive cells, a reduction of more than 10-fold was observed for cells that were grown in the presence of 50 nM silvestrol (Figure 5C).

These data indicate that treatment of HEV-replicating cells with 2 nM or 50 nM silvestrol leads to a decrease in the intracellular amount of HEV capsid protein and reduces the number of HEV-infected cells.

### 3.5. Increased Amount of HEV-Specific RNA upon Silvestrol Treatment Is Not Due to Augmented Genome Replication

The experiments described above (Figure 3) showed that treatment of HEV-replicating A549 cells with the lower concentration of silvestrol (2 nM) leads to an increase of intracellular HEV-specific transcripts after 24 h and 48 h, although the amount of released infectious viral particles is significantly reduced. Several reasons, such as impaired release, decreased degradation, or increased replication can be causative for the elevated amount of HEV-specific transcripts.

**Figure 6:**
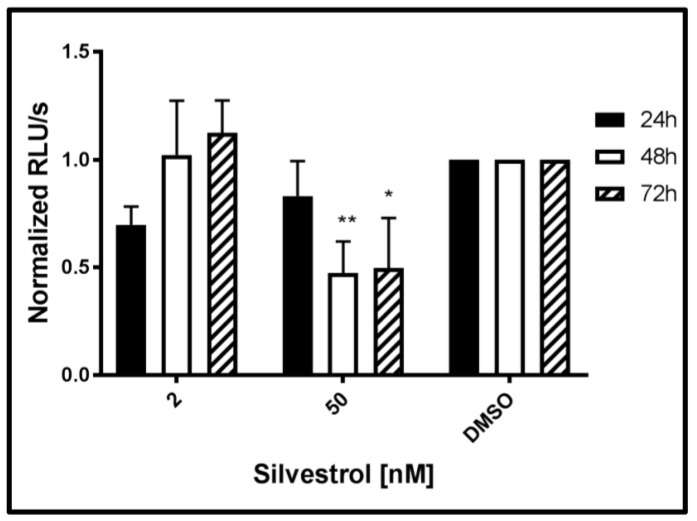
Effect of silvestrol on the genome replication of HEV genotype 3 strain p6Kernow *Gaussia* luciferase reporter constructs in A549 cells. Intracellular luciferase activity measured in RLU/s normalized to total protein amount after silvestrol treatment (2 and 50 nM) compared to the DMSO-treated cells. *n* = 3 for all experiments; * *p* < 0.05, ** *p* < 0.01.

To investigate whether silvestrol additionally affects viral genome replication directly, a reporter genome was used (HEV3 p6GLuc). To exclude that a potential interference of silvestrol with the release of *Gaussia* luciferase (GLuc) affects the results, luciferase activity was determined in the cellular lysates. Measurement of the luciferase activity after silvestrol treatment for 24 h, 48 h, and 72 h revealed that an increase in the genome replication could not be observed for any of the applied silvestrol concentrations, as evidenced by the decreased luciferase activity, when compared to the control, but rather the opposite was the case. For the lower concentration (2 nM silvestrol), an initial inhibitory effect on the viral replication disappeared after treatment for 72 h. In contrast to this, for 50 nM silvestrol after 24 h, almost no effect was observed, while after 48 h and 72 h, a significant reduction was found. These data argue against the hypothesis that the elevated levels of HEV-specific RNA observed after treatment with lower concentrations of silvestrol are due to the positive effect of silvestrol on HEV-replication (Figure 6).

This corroborates the hypothesis that the elevated intracellular level of HEV-specific RNA in silvestrol-treated cells is primarily due to an impaired viral morphogenesis/-release and is not caused by a stimulatory effect on HEV genome replication, since only inhibitory effects are measurable.

### 3.6. The Major Vault Protein (MVP) Was Identified as Antiviral Host-Factor Being Affected by Silvestrol

**Figure 7:**
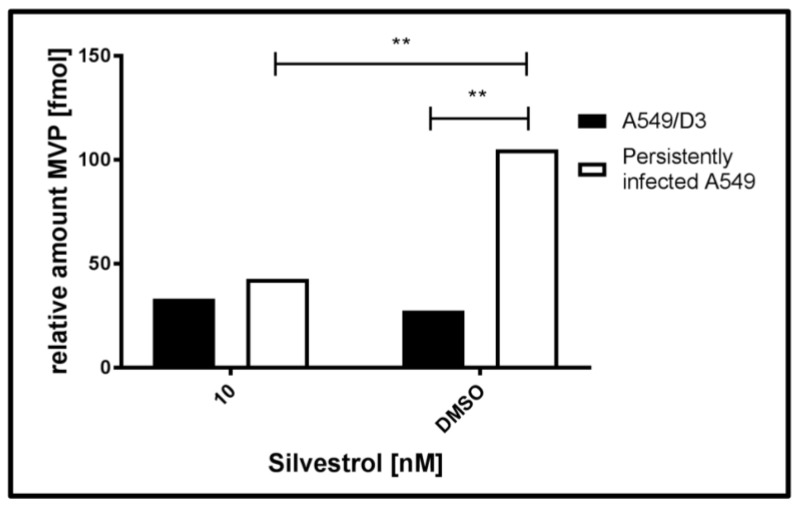
Induction of MVP expression upon HEV infection and subsequent cytosolic decrease by 10 nM silvestrol treatment (24 h) as measured by ultradefinition mass spectrometry (UDMS^E^) and depicted in Table 1. *n* = 3; ** *p* < 0.01.

As indicated by the data described above, silvestrol has an inhibitory effect on the HEV life cycle. Besides that, it has to be asked whether the activity of antiviral mechanisms are affected by silvestrol. To address this, A549/D3 and persistently HEV-infected A549 cells were cultured in the presence or absence of silvestrol. An intermediate concentration of silvestrol (10 nM, treatment lasting for 24 h), where no effect on HEV was present has been chosen. It was hypothesized that this could be due to the decreased antiviral activity that is caused by silvestrol treatment. Cytosolic proteins were extracted and an in-solution protein digestion with subsequent LC/MS (using UDMS^E^ [43]) analysis was performed to identify antiviral host-factors being inhibited by silvestrol. Table 1 shows that the major vault protein (MVP) was unambiguously identified in all of the samples and was proven to be strongly regulated, when HEV-positive (H) and negative cells (D3), and treated (+) and untreated cells (−) were compared. Figure 7 displays a graphical representation of the data depicted in Table 1.

The data shown in Table 1 and in Figure 7 demonstrate that the amount of MVP in the cytoplasm of untreated, persistently HEV-infected A549 cells (H−) is significant higher, when compared to the HEV-negative, untreated control (D3−). In the case of silvestrol-treated cells, however, a significant reduction of MVP in the cytoplasm was observed for the HEV-positive cells (H+), leading to an amount of MVP that is comparable to the amount of silvestrol treated HEV-negative cells (D+).

In a recent study [35], it was described that hepatitis C virus, vesicular stomatitis virus, and influenza A virus induce MVP expression. In turn, MVP affects viral replication by triggering the expression of type-I IFNs.

**Figure 8:**
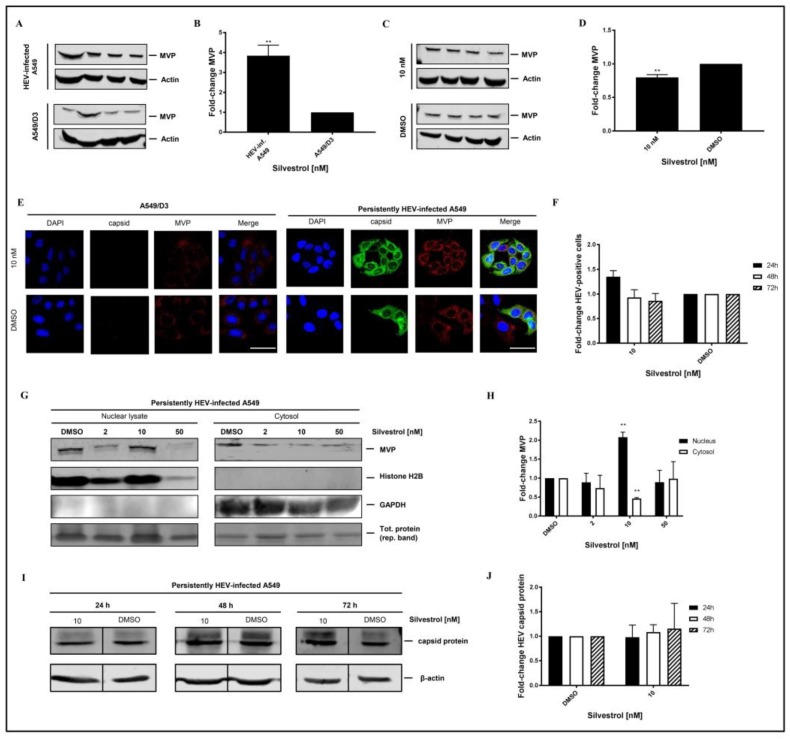
MVP is upregulated in HEV-infected A549 cells and is downregulated and translocated to the perinuclear region upon 10 nM silvestrol treatment. (**A**) Western Blot analysis of cellular lysates derived from persistently HEV-infected A549 cells as compared to A549/D3. An MVP-specific antibody was used for detection, detection of beta-actin served as loading control. (**B**) Shows the corresponding quantification of (**A**) normalized to beta-actin. *n* = 4; ** *p* < 0.01. (**C**) Western Blot analysis of cellular lysates derived from persistently HEV-infected A549 cells treated with 10 nM silvestrol for 24 h. Treatment with DMSO served as a control. (**D**) Shows the corresponding quantification of (**C**) normalized to beta-actin. *n* = 4; ** *p* < 0.01. (**E**) A549/D3 and persistently HEV-infected A549 cells were seeded on cover-slips and treated with 10 nM silvestrol for 24 h. Nuclei were stained with DAPI (blue), HEV capsid protein-specific antibodies (green) and MVP-specific antibodies (red) were used for detection. (**F**) Comparison of the proportion of HEV capsid protein expressing cells after treatment with 10 nM silvestrol depicted in (**E**) to the respective DMSO control; *n* = 3, scale bar = 40 µm (**G**) Western Blot analysis of nuclear lysates and cytosolic protein fraction after subcellular fractionation of HEV-infected A549 cells treated with silvestrol; Histone H2B and ERK1 served as control for nuclei and cytoplasm. (**H**) Normalized Quantification of MVP detected in (**G**); *n* = 3; ** *p* < 0.01. (**I**) Representative Western Blot of cellular lysates derived from cells treated with 10 nM silvestrol. DMSO treatment served as control. For detection, a capsid-protein specific antiserum and a beta-actin-specific antibody for loading control were used. (**J**) Relative quantification of Western Blot band intensities of (**I**) normalized to intracellular beta-actin levels, referred to the DMSO control; *n* = 3.

Western blot analysis using an MVP-specific antibody of cellular lysates that is derived from non-infected A549/D3 cells and from persistently HEV-infected A549 cells confirmed the significant higher amount of MVP in HEV-positive cells (Figure 8A,B). However, Western blot analyses of total cellular lysates derived from silvestrol-treated and untreated HEV-replicating cells only show a moderate decrease on the total amount of MVP (Figure 8C,D). To further address MVP’s subcellular localization under silvestrol-treatment, immunofluorescent stainings (Figure 8E), and subcellular fractionations of persistently HEV-infected A549 cells with subsequent Western Blot analysis and respective quantifications were performed (Figure 8G,H), including a broader range of concentrations for fractionations, matching the initial experimental setup. Proof of a lacking antiviral activity of 10 nM silvestrol-treatment over the course of 72 h is depicted as quantification of HEV capsid protein expressing cells for treated and non-treated, HEV-infected A549 cells (Figure 8F), as well as representative Western Blots and respective quantifications (Figure 8I,J).

On the first glance, the results displayed in Figure 8B seem to be in contrast to the data obtained by MS. However, it has to be considered that the MS data indicate that the amount in the cytoplasm was reduced.

To further investigate this, confocal immunofluorescence microscopy was performed. Confocal immunofluorescence microscopy, using an MVP-specific antibody, of HEV-positive and HEV-negative cells that were or were not subjected to silvestrol treatment solves this seeming contradiction. The immunofluorescence confirms the increase in the amount of MVP in persistently HEV-infected A549 cells as compared to the HEV-negative control cells. The immunofluorescence microscopy further demonstrates that in HEV-positive, silvestrol-treated cells MVP change the subcellular localization. In the case of the untreated cells, a cytoplasmic localization is observed; in case of the silvestrol-treated cells, MVP displays a perinuclear, dot-like staining (Figure 8E). This reflects that the decreased MVP concentration that was observed in the mass spectrometry analysis of the cytosolic fraction is primarily due to the translocation from the cytoplasm to the perinuclear membrane and to a minor part due to the decreased total amount. In addition, subcellular fractionation experiments of cells that are treated with 2 nM, 10 nM, and 50 nM for 24 h were performed. Nuclear lysates and cytoplasm were analyzed by Western Blotting using an MVP-specific antibody (Figure 8G). The quantification of the Western Blots (Figure 8H) shows that the amount of MVP in the cytoplasm is significantly decreased, while it is significantly increased in the nuclear lysate at 10 nM. In accordance to the observed withdrawal of MVP from the cytoplasm, resulting in the loss of its antiviral activity for cells that are treated with 10 nM silvestrol, the quantification of the immunofluorescence staining with the core-specific antiserum (Figure 8E,F) shows no significant reduction in the number of HEV positive cells for 10 nM silvestrol, which stands in contrast to the significant reduction that was observed for 2 nM and 50 nM treated cells (Figure 5B,C). This was also confirmed by Western Blot analysis of cellular lysates derived from cells treated with 10 nM silvestrol for 24 h, 48 h, and 72 h using a core specific antiserum. In contrast to the observed reduction in the amount of intracellular HEV capsid protein observed for cells treated with 2 nM and 50 nM silvestrol (Figure 4B,C), this was not observed for cells that were treated with 10 nM silvestrol (Figure 8I,J).

These data demonstrate that the direct antiviral effect of silvestrol on the translation of the viral RNA is embedded in the silvestrol-dependent modulation of an antiviral activity.

## 4. Discussion

Silvestrol inhibits the eukaryotic initiation factor 4A (eIF4A), and it thus affects the protein synthesis from some 5′-m7GTP-capped mRNAs. As HEV comprises such capped RNA structures in its (+)-strand ssRNA genome and in the bicistronic RNA subgenome, the effects of silvestrol on the HEV life cycle were investigated. Antiviral effects of silvestrol are not unprecedented. In recent studies, an inhibitory effect of silvestrol on the Ebola virus (EBOV), Picorna- Corona-, and Zika virus was observed [26,27,28].

For HEV, the presented results show a significant reduction of infectious HEV particle release into the culture supernatant of persistently HEV-infected A549 cells upon treatment with silvestrol at concentrations of 2 nM and 50 nM.

Comparing the amount of released viral genomes to the number of infectious viral particles shows that the initial decrease in the amount of released viral genomes (24 h and 48 h) at 2 nM silvestrol disappears after 72 h. In contrast to this, the number of released infectious viral particles shows a persistent reduction over time.

Since only a mild cytotoxic effect is present at early time points of treatment with silvestrol at a concentration of 2 nM and not at later time points, where the major effect on viral particle or HEV-carrying exosomes [18,21] release takes place, this effect cannot be due to a rupture of cells, but it may be caused by extracellular vesicles being released by the cells, thereby releasing partially unspecific intracellular components, such as proteins and RNA, to the extracellular space, as seen in several studies [46,47] and as reported for the release of e.g., Hepatitis C virus (HCV) RNA into the cell culture medium via exosomes [48].

For 50 nM silvestrol, a strong inhibition of the HEV life cycle can be observed. This is reflected by a strong decrease in the number of released infectious viral particles and by the immunofluorescence microscopy showing less HEV-positive cells (reflecting the impaired spread of HEV, due to the reduced number of infectious particles), while these HEV-positive cells show a strongly reduced capsid protein–specific signal (reflecting the impact on translation of viral proteins). A recent study highlighted that there are three forms of the HEV capsid protein that are present in infected cells, being part of different stages of the viru’s life cycle [49]. We observe an overall decrease of signals in Western Blot analyses leading to comparable reductions of these forms, respectively. Since HEV genomic replication is not significantly inhibited by silvestrol over the course of up to 72 h, it is further highlighted that the compound mainly triggers structural protein biosynthesis, thereby hindering capsid formation and viral particle release, rather than inhibiting the synthesis of viral genomes for its progeny.

The viability assays show a decrease in the redox-metabolism at 50 nM (factor 2). However, when compared to the effect on the amount of released viral particles (factor 250), this effect on redox-metabolism is much smaller than the effect on HEV and there is no significant effect on the cellular integrity (LDH assay), confirming the specificity of the observed effect. It has to be noted that the observed effect of silvestrol on the redox-metabolism of A549/D3 cells that was observed in this study partially differs from a previous one [28], observed for A549 cells. Moreover, DAPI staining shows no formation of fragmented nuclei, which would indicate apoptosis.

The observed antiviral effect of silvestrol seems to be complex. On the one hand, there is a direct effect of silvestrol on the translation of viral proteins, as reflected by the decrease in the amount of capsid protein. On the other hand, it should be considered that silvestrol, as an inhibitor of eIF4A, also affects the translation and activity of a variety of host-factors. These factors could include RNases and proteasomal, lysosomal, or autophagosomal proteins. If such activities, triggering the degradation of viral genomes or viral proteins, are impaired, a stabilization of i.e., viral genomes is the consequence, which could compensate for the inhibitory effects of silvestrol on other parts of the viral life cycle.

In this context, host-factors may play a crucial role normally acting inhibitory on HEV themselves under natural conditions. MVP was identified as a host-factor, which is strongly upregulated in HEV-positive cells. It was described recently that Hepatitis C virus, Vesicular stomatitis virus, and Influenza A virus induce MVP expression. In turn, MVP affects viral replication by e.g., triggering the expression of type-I IFNs [35]. These previous studies show that MVP is an important protein in regulating and propagating the innate immune-response on a cellular level and highlight MVP’s function of being part of a virus-inducible host-defense mechanism. By an intermediate concentration of silvestrol (10 nM), the amount of MVP in HEV-positive cells is moderately, but significantly, decreased. However, the subcellular localization is strongly affected by silvestrol, leading to a strong decrease of the MVP levels in the cytosol, as evidenced by mass spectrometry. This is due to a translocation of MVP from the cytoplasm to the perinuclear region, as shown by immunofluorescence microscopy. To set the observations that were made in LC/MS and immunofluorescence in a broader context, we prepared nuclear lysates and cytoplasms from cells treated with 2 nM, 10 nM, and 50 nM silvestrol, and analyzed these fractions by Western Blot, while using an MVP-specific antibody. DMSO treated cells served as a control. These experiments confirm the mass spectrometry and immunofluorescence results that were obtained for 10 nM, as these data show that there is a significant decrease of MVP in the cytoplasm and a significant increase of MVP in the nuclear and perinuclear fraction at 10 nM, as compared to the DMSO control. For 2 nM and 50 nM silvestrol, there is no significant change, as compared to the DMSO control. This translocation prevents that MVP fulfils its cytosolic signal transduction on e.g., the induction of type I interferons, which has been described as part of its antiviral response to HCV [35,50] or ZIKV [28]. This illustrates that the observed effects of silvestrol on the HEV life cycle reflect overlapping effects with different kinetics of silvestrol on HEV and on host cell-factors, which depends on the concentration of silvestrol and the duration of silvestrol treatment.

To consider silvestrol as a novel treatment for HEV infection in human patients, more research needs to be done in this field. Furthermore, one could think of a combination treatment of silvestrol and e.g., Ribavirin, which is the common treatment for HEV-infected individuals, since, in this case, both viral replication [51,52], as well as viral protein translation can be inhibited. As our study is limited on a genotype 3i HEV strain and on the HEV genotype 3 p6Kernow strain, it may be useful to check for other genotypes being affected by silvestrol, as well as elucidating, whether or not MVP behaves in a similar manner. Hereby, a true broad-spectrum, clinical usage of the compound can be considered.

## 5. Conclusions

Our study identifies silvestrol as an inhibitory agent against HEV with a significant reduction on viral particle release and viral protein biosynthesis. We furthermore identified MVP as an antiviral host-factor being affected by the natural compound, leading to a complex interplay between the direct effects on the viral life cycle and the effects on antiviral activities of the host-cell. When considering this complex interplay between silvestrol’s action on HEV and host-factors, it can be stated that silvestrol exerts a strong antiviral effect on HEV, as reflected by the robust reduction of the amount of released viral particles. As HEV is a virus with a short 5′-UTR without complex secondary RNA structures, these data provide evidence for a broader spectrum of silvestrol’s antiviral usage.

## Figures and Tables

**Table 1:** Identification and quantification of MVP by UDMS^E^. Cytosolic protein levels of Major Vault Protein (MVP) (UniProt accession number, number of tryptic digests identified by MS, number of unique peptides identified by MS and confidence score listed in column 1 to 5) of 10 nM treated or non-treated A549/D3 or persistently HEV-infected A549 cells (column 8 and 9) with annotated *p*-values (column 6) and fold-change (column 7). Row 4: A545/D3 untreated vs. A549/D3 treated; Row 6: infected A549 untreated vs. infected A549 treated; Row 8: A549/D3 untreated vs. infected A549 untreated; **Row 10**: A549/D3 treated vs. infected A549 treated. *n* = 3.

Protein Identification					Protein Expression Level			
Acc.No.	Description	P	UP	CS	Anova (p)	FC	Amount (fmol)	
							D3−	D3+
Q14764	Major vault protein	10	5	67.4	0.852	1.2	27.6	33.2
							H−	H+
					0.015	2.5	105.1	42.7
							D3−	H−
					0.021	3.8	27.6	105.1
							D3+	H+
					0.463	1.3	33.2	42.7

Acc.No.: UniProt acession number; P: number of tryptic peptides identified by MS; UP, number of unique peptides identified by MS; CS: confidence score; FC: fold change; D3: non-infected A549D3 cells; H: HEV infected A549 cells; − not treated; + treated with silvestrol.
